# Rapid Hydrogen Peroxide release from the coral *Stylophora pistillata* during feeding and in response to chemical and physical stimuli

**DOI:** 10.1038/srep21000

**Published:** 2016-02-15

**Authors:** Rachel Armoza-Zvuloni, Avi Schneider, Daniel Sher, Yeala Shaked

**Affiliations:** 1 Interuniversity Institute for Marine Sciences, Eilat, 88103, Israel; 2 The Fredy & Nadine Herrmann Institute of Earth Sciences, The Hebrew University, Jerusalem, 91904, Israel; 3 Department of Marine Biology, Charney School of Marine Sciences, Haifa University, Haifa, Israel

## Abstract

Corals make use of different chemical compounds during interactions with prey, predators and aggressors. Hydrogen Peroxide (H_2_O_2_) is produced and released by a wide range of organisms as part of their defense against grazers or pathogens. In coral reefs, the large fluxes and relatively long half-life of H_2_O_2,_ make it a potentially important info-chemical or defense molecule. Here we describe a previously unstudied phenomenon of rapid H_2_O_2_ release from the reef-building coral *Stylophora pistillata* during feeding on zooplankton and in response to chemical and physical stimuli. Following stimuli, both symbiotic and bleached corals were found to rapidly release H_2_O_2_ to the surrounding water for a short period of time (few minutes). The H_2_O_2_ release was restricted to the site of stimulus, and an increase in physical stress and chemical stimuli concentration resulted in elevated H_2_O_2_ release. Omission of calcium (a key regulator of exocytotic processes) from the experimental medium inhibited H_2_O_2_ release. Hence we suggest that H_2_O_2_ is actively released in response to stimuli, rather than leaking passively from the coral tissue. We estimate that at the site of stimulus H_2_O_2_ can reach concentrations potentially high enough to deter predators or motile, potentially pathogenic, bacteria.

Cnidarians (including corals, sea anemones, jellyfish and hydras) are soft-bodied, morphologically simple, and predominantly sessile organisms. Since their early emergence (~580 million year ago) cnidarians have become abundant in all oceans and typically form the foundation of coral reefs^1^,^2^. Much of their success is attributed to their ability to chemically interact with predators, prey, aggressors, microbial pathogens and fouling organisms^3^,^4^. The numerous chemicals cnidarians employ in these interactions can be broadly divided into secondary metabolites and toxic or antimicrobial peptides and proteins^5^. Secondary metabolites, often possessing antimicrobial, antifouling, or predator deterrence properties, have been characterized mostly in soft corals^4^,^5^. Secondary metabolites are typically stored in epidermal cells and are released regularly through diffusion or following tissue injury^3^,^4^,^5^. In contrast, many toxic peptides and proteins are associated with cnidarian venom, which is injected through specialized stinging cells (known as nematocytes or cnidocytes)^3^,^6^. When an appropriate physical and chemical stimulus is identified, the cnidocytes discharge at high acceleration, punching into the prey or predator and injecting venom using a highly specialized microscopic secretory structure (the cnidocyst)^6^. Nevertheless, several peptide and protein toxins, with possible roles in predation or digestion, are stored in, and secreted from other cells or tissues^7^,^8^,^9^. Among cnidarians, the chemical ecology of stony corals has been less studied^5^,^10^. Yet, like other cnidarians with limited mobility, stony corals may also rely on secondary metabolites, peptides and proteins for chemical defense and prey capture.

Hydrogen peroxide (H_2_O_2_) is a common metabolite of aerobic reactions, produced mostly during respiration and photosynthesis^11^. At low concentrations H_2_O_2_ can serve as a signaling molecule but at high concentrations it may cause direct oxidative damage to various cellular components (including proteins, lipids and DNA)^11^. H_2_O_2_ may also initiate lethal cellular cascades such as lipid peroxidation and apoptotic cell death^11^,^12^ and is often used in interactions between organisms, as a grazer and pathogen deterrent^13^. Rapid release of extracellular H_2_O_2_ and other oxidants, known as an oxidative burst, is used by microorganisms, plants and mammalian immune cells as a defense against pathogenic bacteria and fungi^11^,^14^.

In corals, H_2_O_2_ and other active oxygen molecules, commonly termed reactive oxygen species (ROS), have been studied in the context of the symbiosis between the coral host and its resident algae – the zooxanthellae^15^. While ROS are produced by several metabolic pathways in the coral holobiont (i.e. coral, zooxanthellae and associated microbes^16^), the zooxanthellae’s photosynthesis is considered to be the major source of ROS^15^,^17^. The flux of ROS is normally regulated, through detoxification, by a range of antioxidant molecules (such as the enzymes catalase and peroxidase) synthesized by the different members of the coral holobiont^18^. Under severe environmental stress ROS may accumulate in the coral tissue and induce coral bleaching^15^,^17^,^18^,^19^ (loss of symbiotic algae).

Recently we identified and characterized a flow-induced long term release of H_2_O_2_ and superoxide (O_2_^−^) from the coral *Stylophora pistillata*^20^,^21^,^22^, occurring on a time-scale of hours. Here, we explore another yet unstudied phenomenon, namely short term H_2_O_2_ release from *S*. *pistillata* which occurs on time-scales of minutes in response to various stimuli. In incubation experiments with healthy and bleached coral fragments we documented rapid H_2_O_2_ release during feeding on zooplankton prey and in response to physical and chemical stimuli. In the absence of calcium, a factor known to regulate exocytotic processes, we observed no H_2_O_2_ release. By applying localized stimuli and frequent sampling we found that the response is limited to the site of stimulus (rather than whole colony response) and that its duration is limited to few minutes.

## Results

### H_2_O_2_ is actively released during feeding of coral on zooplankton

**Figure 1:**
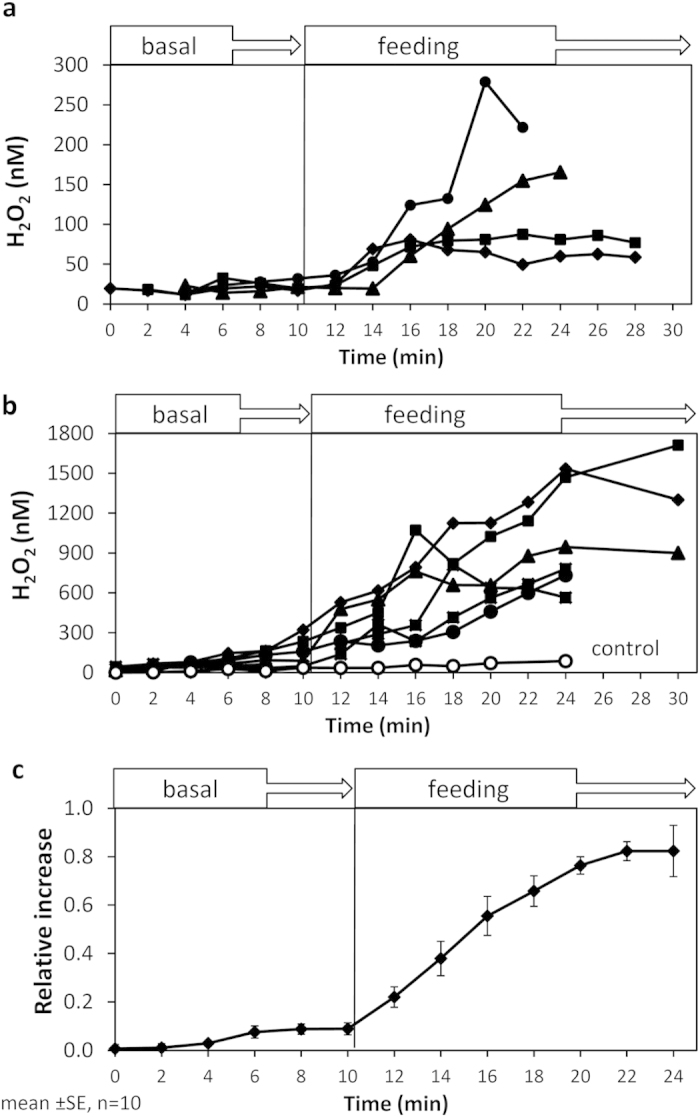
Rapid H_2_O_2_ release from *Stylophora pistillata* during feeding. (**a**,**b**). Changes in H_2_O_2_ concentration before food was supplied (basal) and during feeding in ten individual coral fragments. Note the high variation between individuals (panels a and b have a different y-axis scale). Release of H_2_O_2_ from control untreated corals is shown in panel b as mean ± SE (n = 6). (**c**) Summary of all experiments presented as mean ± SE of the change in H_2_O_2_ with time relative to the maximal concentration in each experiment.

We probed for H_2_O_2_ release during feeding by adding *Artemia salina* nauplii to the water surrounding *S. pistillata* coral fragments suspended in 100 mL glass beakers (see materials and methods and Fig. S1 for a detailed description of the experimental setup). Visual observations confirmed that the corals responded immediately to the addition of nauplii by capturing and paralyzing the prey with their hunting tentacles (Fig. S1c). Following the addition of the nauplii, H_2_O_2_ concentrations increased around all coral fragments and kept increasing throughout the experiment, reaching an average concentration of 850 ± 600nM (mean ± SD, n = 10). Significant variation was observed among individual coral fragments in the amount of H_2_O_2_ that accumulated in the beaker, which we refer to as H_2_O_2_ released hereafter (Fig. 1a,b). Searching for a way of representing the averaged behavior of all corals, we chose to normalize all H_2_O_2_ measurements of an individual experiment to its maximal H_2_O_2_ concentration. Presented in this manner, the results suggest relatively uniform and consistent H_2_O_2_ release kinetics (Fig. 1c). Prior to prey addition and in control experiments with untreated (unfed) corals, we observed slow release of low levels of H_2_O_2_ (Fig. 1a,b). This slow H_2_O_2_ release, termed here basal release, reflects the coral response to the water movement (stirring) as documented in Armoza-Zvuloni and Shaked^22^. Given this background, the statistical tests of the coral response to a specific stimulus have to take into account the basal H_2_O_2_ release. Comparisons between few experimental sections, different stimuli, media composition, etc., were made by comparing their ΔH_2_O_2,_ which is the change in H_2_O_2_ between the start and the end of each experimental section (see methods). Here, addition of nauplii resulted in ΔH_2_O_2_ that were significantly higher than basal ΔH_2_O_2_ (p < 0.01, paired t-test; Fig. 1a–c). When measuring H_2_O_2_ concentrations in coral-free seawater containing similar numbers of live, homogenized or paralyzed nauplii (i.e. gently collected from the corals before being eaten) all tests revealed low and stable H_2_O_2_ signals verifying that the released H_2_O_2_ did not originate from the nauplii themselves (Supplementary Fig. S2).

**Figure 2:**
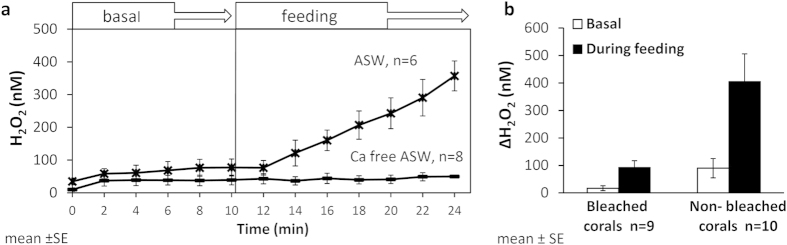
Release of H_2_O_2_ during feeding in Ca free conditions and in bleached *S. pistillata.* Changes in H_2_O_2_ concentrations over time in (**a**) artificial seawater (ASW) with or without Ca and in (**b**) bleached coral fragments compared to non-bleached ones. The white bars in panel b represent the change in H_2_O_2_ concentrations over 10 min, prior to food supply (basal) and during feeding (black bars).

To further link H_2_O_2_ release to the prey capture activity, we inhibited pray capture by using calcium free seawater (Ca^2+^ is required for the activation of the cnidocytes, the coral’s stinging cells^6^). In calcium free artificial seawater (Ca-free ASW) the corals did not catch the prey although the nauplii were swimming next to the coral hunting tentacles. In these experiments, H_2_O_2_ concentrations remained low and constant throughout the experiment (ΔH_2_O_2_ was not different before and following nauplii addition, p > 0.05, paired t-test; Fig. 2a). In the control experiment, using ASW containing Ca, H_2_O_2_ concentrations increased following nauplii addition to the beaker (ΔH_2_O_2_ was significantly different, p < 0.01, paired t-test; Fig. 2a) and corals were observed preying upon the swimming *Artemia salina* nauplii.

To examine the possible involvement of the coral symbiotic algae (the zooxanthellae) in the rapid H_2_O_2_ release we conducted additional feeding experiments with bleached (algae-free) corals. We observed prey capture by the bleached corals and detected H_2_O_2_ release in response to the feeding (Fig. 2b). The release of H_2_O_2_ (ΔH_2_O_2_) from bleached corals during feeding was significantly higher than their basal H_2_O_2_ release (p < 0.01, paired Wilcoxon’s test) but lower than that from non-beached corals (p < 0.05, independent samples t-test; Fig. 2b). Here, again, high variation among individual coral fragments was observed in the amount of H_2_O_2_ released, as shown in Supplementary Fig. S3.

### Rapid release of H_2_O_2_ in response to chemical stimuli

**Figure 3:**
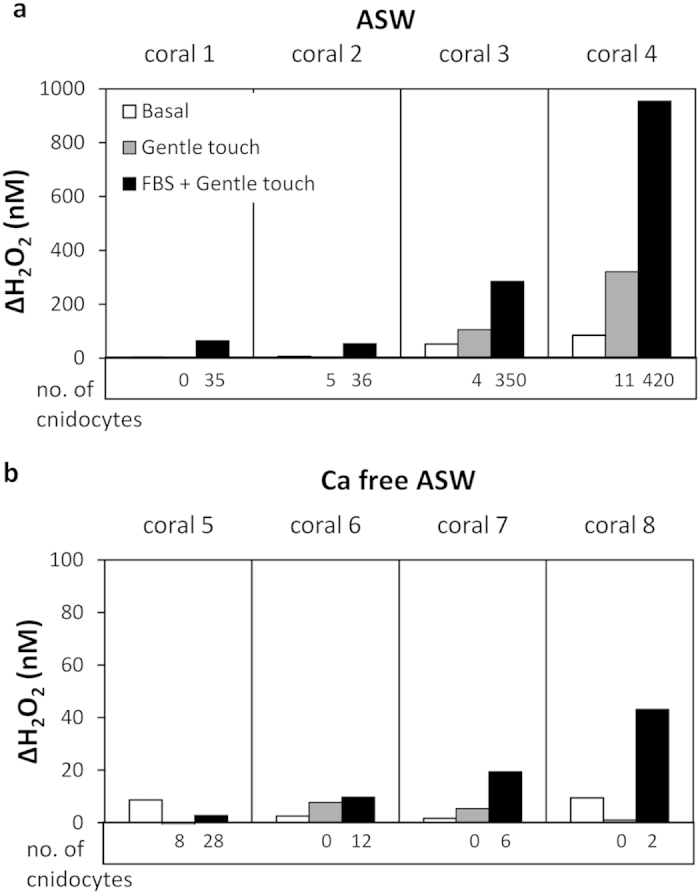
Release of H_2_O_2_ and cnidocyte discharge in response to chemical and gentle physical stimuli (FBS) in ASW (**a**) and Ca-free ASW (**b**). H_2_O_2_ concentrations were followed in individual coral fragments prior to and following two stimuli: a gentle physical stimulus with a Pasteur pipette and a gentle physical stimulus with an FBS covered Pasteur pipette. The bars represent the change in H_2_O_2_ concentrations during ten minutes prior to stimuli (basal, white bars) and then during ten minutes following each stimulus (pipette only, grey bars; FBS covered pipette, black bars). The number of cnidocytes discharged by each stimulus is listed below each respective bar.

During prey capture corals discharge cnidocytes from their hunting tentacles. This discharge is known to be best activated by a combination of physical and chemical stimuli^6^. To determine whether there is a possible link between the discharge of cnidocytes and H_2_O_2_ release, we conducted an experiment utilizing known triggers of the coral’s stinging mechanism, namely gentle physical stimulus with Fetal Bovine Serum (FBS). As a control we tested the coral response to gentle physical stimulus without FBS. Similar to other experiments, prior to the treatments we observed some basal H_2_O_2_ release (Fig. 3a, white bars). Next, following the gentle physical stimulus without FBS, we measured a slight increase in H_2_O_2_ release by two of the four coral fragments tested (Fig. 3a, grey bars) and we recorded a minor discharge of cnidocytes (Fig. 3a, numbers below the bars). Finally, we measured high H_2_O_2_ release in response to FBS, significantly higher than H_2_O_2_ release in both basal and gentle physical stimulus (p < 0.01 and p < 0.05 respectively, one way Repeated Measures ANOVA and Bonferroni’s test for post hoc comparison; Fig. 3a). While we attribute this increase to the combination of chemical and physical stimuli, we cannot rule out the possibility of a delayed increase in H_2_O_2_ release in response to the physical stimulus. FBS resulted also in strong cnidocyte discharge in all corals (~40 fold increase on average; Fig. 3a, black bars and Fig. S4). When conducting the same experiment in a calcium free artificial seawater we found that H_2_O_2_ did not increase significantly following all treatments (p > 0.05, one way Repeated Measures ANOVA) and that the number of discharged cnidocytes increased only slightly in response to FBS (Fig. 3b, note the different scale of the y-axis; Fig. S4).

### Rapid release of H_2_O_2_ in response to physical stimulus

**Figure 4:**
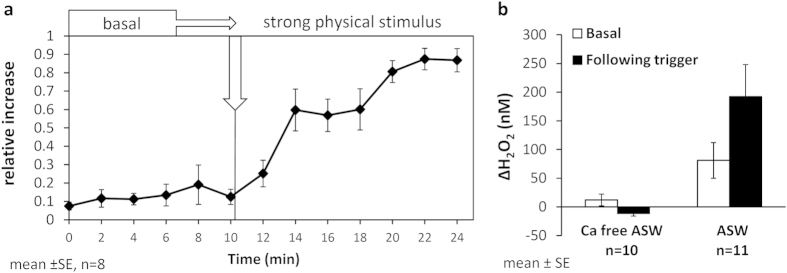
Rapid H_2_O_2_ release from *S. pistillata* following strong physical stimulus. (**a**) Relative increase in H_2_O_2_ before applying the stimulus (basal) and following strong physical stimulus. (**b**) Similar experiments were also run in artificial seawater (ASW) and in Calcium-free ASW (panel b). The bars in panel b represent the change in H_2_O_2_ concentrations over 10 min, prior to stimulus (basal; white bars) and following it (black bars).

We probed for H_2_O_2_ release during strong physical stimulus using a glass Pasteur pipette (Fig. S1b). The pressure applied with the pipette was ~13 fold higher than that applied during the gentle stimulus and the contact time was longer (see materials and methods). In response to this treatment the coral polyps that were initially extended retracted. This physiological response was accompanied by a significant increase of H_2_O_2_ concentrations following the stimulus (p < 0.01, paired t-test; Fig. 4a, Supplementary Fig. S5). As in the feeding experiments, some basal H_2_O_2_ release was recorded prior to the physical stimulus (Fig. 4a).

In order to determine whether the observed H_2_O_2_ release is an active process or a passive leakage from damaged tissue we repeated this experiment in artificial seawater (ASW) with and without calcium, since Ca is known to be required for many exocytotic processes besides cnidocyte discharge. The results show that in the absence of Ca, H_2_O_2_ concentration did not increase, prior to or in response to the strong physical stimulus (Fig. 4b and individual fragments in Fig. S6). In the presence of Ca, a statistically significant enhancement of H_2_O_2_ release was observed in response to the strong physical stimulus (p < 0.01, paired t-test; Fig. 4b). In some of the experiments with ASW we observed high increase in H_2_O_2_ concentration, which probably resulted from the coral transfer between beakers, an agitation we previously reported to influence H_2_O_2_ release^22^.

In a separate experiment, we tested the hypothesis that a compound or enzyme producing H_2_O_2_, rather than H_2_O_2_ itself, is released from the cell. We found that accumulation of H_2_O_2_ occurred only in the presence of the coral and not in water retrieved from the coral’s vicinity after the stimulus was administered (Supplementary Fig. S7), thus supporting direct H_2_O_2_ release from the coral.

### Active release of H_2_O_2_ is limited to the site of stimulus

**Figure 5:**
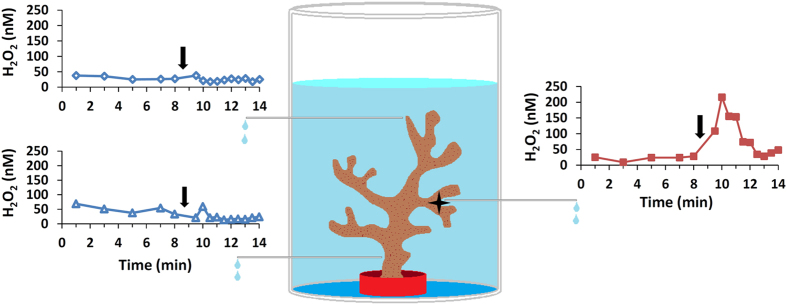
Schematic experimental setup and an example data set from the localized experiments. Water samples were collected with narrow tubing from three sites next to the coral surface for H_2_O_2_ measurements. After eight minutes of sample collection (black arrows), the coral was applied with strong physical stimulus, at only one site (black star). The localized stimulus led to an increase in H_2_O_2_ concentrations at the site of stimulus (red squares), but not at the other sites (empty triangles and diamonds).

In order to determine whether H_2_O_2_ is released only at the site of stimulation or further away in the colony as well, we simultaneously measured the concentrations of H_2_O_2_ at the site of stimulation and at two distant locations (Fig. S1d). The results show that local strong physical stress leads to H_2_O_2_ release only from the site of stimulus (Fig. 5; labeled with a star). At the same time, H_2_O_2_ concentrations did not increase in other parts of the colony (p > 0.05 for both controls, one way Repeated Measures ANOVA and Bonferroni’s test for post hoc comparison were used here and below). This localized H_2_O_2_ release occurs rapidly (within 1.5 min) and lasts for only 4 min. In this experiment, in the absence of stirring, no basal H_2_O_2_ release was observed prior to the localized stress.

**Figure 6:**
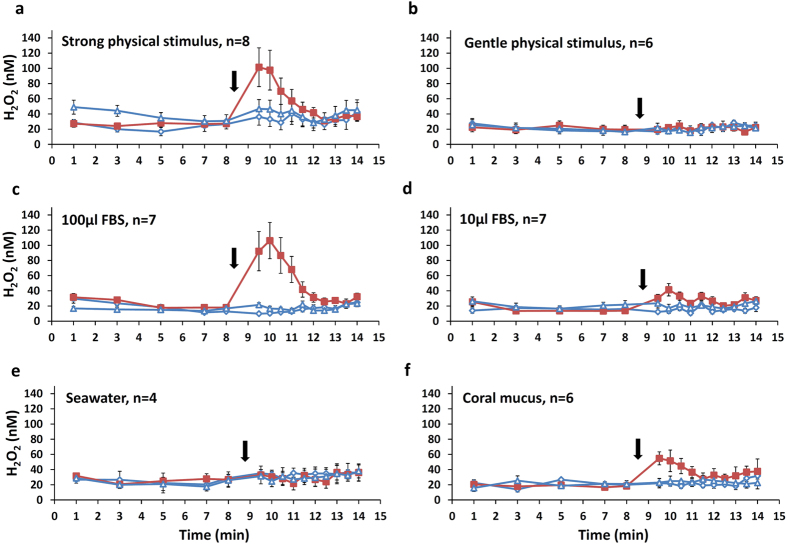
Rapid and localized release of H_2_O_2_ following different treatments. H_2_O_2_ concentrations were measured at three locations near the coral surface. The corals were treated at only one location (red squares) while at two locations no treatment was applied (empty triangles and diamonds). Treatment was applied at eight minute (black arrows) and the coral response to stimuli examined until min 14. The treatments included strong and gentle physical stimuli (**a**,**b**), Fetal Bovine Serum at different concentrations (FBS, **c**,**d**), seawater (**e**) and coral mucus (**f**). Values are means (±SE) of 4–8 repeats as labeled.

In a series of experiments utilizing this setup, strong physical stimulation elicited prominent H_2_O_2_ release (p < 0.01) while gentle physical stimulus did not result in significant release (p > 0.05; Fig. 6a,b). Gentle application of 100 μl FBS to the coral surface prompted strong H_2_O_2_ release (6.8 fold increase; p < 0.01) whereas a smaller volume of 10 μl FBS resulted in a more moderate release (2.5 fold increase; p < 0.01; Fig. 6c,d). To ensure that the coral responded specifically to the FBS and not the jet of water from the pipette, we repeated the test with a similar volume of seawater at different temperatures and observed no H_2_O_2_ release (p > 0.05; Fig. 6e; Fig. S8). Interestingly, fresh coral mucus collected from another *S. pistillata* fragment triggered H_2_O_2_ release (p < 0.01) at a magnitude resembling that seen in response to FBS (3 fold increase; Fig. 6f), possibly implying a broad responsiveness of corals to chemical cues. When tested separately, the mucus showed no preexisting concentration of H_2_O_2_. In summary, in this set of experiments the release of H_2_O_2_ was confined both in time and space −H_2_O_2_ concentrations increased only at the site of stimulation and dropped back to background levels within 4–6 minutes.

## Discussion

In this report we present evidence of a rapid release of H_2_O_2_ from the hermatypic (reef-building) coral *Stylophora pistillata* during feeding and in response to chemical and physical stimuli (Figs 1, 2, 3, 4, 6). This release was not seen in the absence of calcium (Figs 2, 3, 4), and was found to be a short lived, local response with an amplitude related to that of the applied stimulus (approximately 4–6 minutes long, Figs 5 and 6, see also below). Our results suggest that this rapid release is an active process, responsive to various stimuli, some of which are considered stressors (e.g., physical stimuli) while others are beneficial to the coral (feeding). In the ensuing discussion we examine the kinetics and concentrations of this process, explore possible regulators and mechanisms of release, and finally speculate on some potential physiological and ecological roles of this phenomenon.

At first glance the kinetics of H_2_O_2_ release in our two experimental setups do not match, with continuous H_2_O_2_ accumulation in the batch experiments (Figs 1 and 4), versus temporally confined H_2_O_2_ release in the localized experiments (Figs 5 and 6). However, this mismatch most likely reflects experimental differences such as mixing (or its absence) and retention (or removal) of high-H_2_O_2_ water, rather than biological differences. Summing up the discrete H_2_O_2_ fractions collected through the tube in the localized experiments, we end up with H_2_O_2_ accumulation patterns that resemble those of the batch experiments (Supplementary Fig. S9). Alternatively, calculating the change in H_2_O_2_ in each time interval of the batch experiments (and accounting for basal H_2_O_2_ release), we end up with H_2_O_2_ release kinetics that resemble those of the localized experiments (Fig. S9). The emerging pattern is hence of an immediate increase in H_2_O_2_ in response to the stimuli (within 1–2 min) that lasts for several minutes (4–6 min).

The H_2_O_2_ concentrations we measured varied between fragments, stimuli, and experimental setup, with maximal accumulated release of ~1600 nM in the feeding batch experiments (Fig. 2). These concentrations are higher than measured in Gulf of Aqaba open waters^23^ or coral reefs^21^, and are comparable to those released from corals subjected to strong stirring^22^. However, our experimental choices of water volume or dripping rate through the narrow tubes (Supplementary Fig. S1) clearly influence the measured H_2_O_2_ concentrations. Furthermore, H_2_O_2_ release rate, the ambient water flow, and the ciliary motion at the coral surface^24^,^25^,^26^, all likely affect the concentration field of H_2_O_2_ surrounding the coral, thus affecting the doses encountered by potential predators and/or pathogenic bacteria. We lack precise measurements of H_2_O_2_ concentrations within the diffusive boundary layer (DBL) of the coral (a few millimeters thick), which are of relevance for invading and resident bacteria. However, we can conservatively estimate these concentrations based on previous measurements showing that the concentrations of oxygen within the DBL are approximately double those measured outside of it^25^,^26^,^27^. Using this approach, concentrations of ~400 nM within the DBL can be expected based on the ~200 nM H_2_O_2_ measured outside the DBL, ~0.2 cm from the coral tissue (Figs 5 and 6). An alternative approach is to estimate the H_2_O_2_ concentration in the BDL by correcting the localized experiment values for the dilution that occurred during sample collection. This calculation, which is detailed in the supplementary file, results in maximal concentrations of ~40 μM H_2_O_2_ within the DBL during the few minutes following stimulus (Supplementary S10). These two estimates provide a range of H_2_O_2_ concentrations (0.4–40 μM) that might be sensed by microorganisms in the coral’s vicinity following a stimulus (see below).

What is the source of the released H_2_O_2_? Many studies have shown that significant amounts of ROS in general, and H_2_O_2_ in particular, are produced as a by-product of photosynthesis by the symbiotic zooxanthellae^15^,^17^,^18^,^19^. In accordance with those studies, we have previously reported that bleached *S. pistillata* do not release H_2_O_2_ in a flow-induced continuous pathway (referred to here as basal release), and have hence suggested that the symbiotic algae are responsible for this basal H_2_O_2_ flux^22^. In contrast, here, feeding experiments with bleached corals revealed rapid H_2_O_2_ release, although to a lesser extent than non-bleached corals (Fig. 2b). Release of H_2_O_2_ has previously been observed from the non-symbiotic gorgonian *Lophogorgia chilensis*^28^, suggesting that in both these cases the symbiotic algae are not the only source for rapidly-released H_2_O_2_. There are several known enzymatic pathways that produce H_2_O_2_ (e.g. NADPH-dependent oxidases and peroxidases), that operate during the oxidative burst responses of plants and animals as part of their defensive measures against pathogenic bacteria^14^. Given the localized, stimulus-specific and rapid nature of the H_2_O_2_ release observed here, we hypothesize that it is formed through a similar enzymatic mechanism. This hypothesis awaits experimental testing.

With regard to the origin of the produced H_2_O_2_, we were able to rule out leakage of cytosol containing H_2_O_2_ from damaged cells since the rapid H_2_O_2_ release requires calcium (Figs 2, 3, 4) and it occurs in response to stimuli other than the physical ones (Figs 1, 2, 6). In addition, no H_2_O_2_ production was observed once the coral was removed from the water (Fig. S6), suggesting that H_2_O_2_ itself is actively released from the coral, rather than H_2_O_2_ producing enzymes. Could the observed H_2_O_2_ be part of the cnidocyst venom, injected in response to the applied stimuli? Indeed, previous studies have shown that the cnidocyst venom or tentacle extract of several sea anemones and jellyfish causes lipid peroxidation and/or increases intracellular reactive oxygen species (as measured by the DCFH-DA method)^29^,^30^,^31^, and it has been hypothesized that this effect can contribute to the toxicity of the venom. On the one hand, our data support this view as they show that both H_2_O_2_ release and cnidocytes discharge are triggered and inhibited by the same factors. On the other hand, the timing and magnitude of H_2_O_2_ release and cnidocytes discharge are quite different (Fig. 3), pointing at a lack of direct link between these processes. A possible indirect link may involve injury of cells adjacent to cnidocysts during their explosive discharge and subsequent H_2_O_2_ leakage. Alternatively, H_2_O_2_ may be actively secreted from the ectoderm, as shown for several toxins from anemones and Hydra^8^,^9^. All in all, given the complexity of the coral holobiont, our results leave many open questions regarding the mechanisms of this rapid H_2_O_2_ release.

What biological roles could the H_2_O_2_ actively released from the corals perform in nature? While often thought of as a killing compound involved in organism’s defenses against bacteria and cancer cells, H_2_O_2_ is in fact a “Jekyll and Hyde” molecule, which at sub-lethal concentrations can act as an infochemical used for intra- and inter-organism signaling^32^,^33^. Our estimated H_2_O_2_ concentrations that range between 0.4–40 μM are lower than those typically needed to kill many bacteria, including coral pathogens (>1 mM)^34^, but are within the micromolar range shown to elicit changes in gene expression as well as negative chemotaxis in bacteria^35^,^36^. Additionally, these concentrations are within the range shown to inhibit the feeding of the amphipod *Gondogeneia antarctica*^13^, but are below those shown to affect the feeding of spiny lobsters^37^. Thus, we hypothesize that the active release of H_2_O_2_ from *Stylophora pistillata,* as well as from other corals^28^ is used to deter (but not kill) predators as well as pathogenic bacteria which might be expected to invade the coral tissue following physical damage. Further research is needed in order to test these hypotheses at ecologically relevant times and doses, and to determine to what extent rapidly-released H_2_O_2_ contributes to the pools of this molecule in the reef at different scales^20^,^21^,^22^,^28^,^38^. Given the importance of chemical cues in signaling the health of coral reefs^39^, and the relatively long half-life of H_2_O_2_ (0.5–1 days)^21^, understanding the factors governing H_2_O_2_ concentrations in coral reefs and their response to natural and anthropogenic interferences may have important applications in reef restoration efforts.

## Materials and Methods

### Coral handling and preconditioning

40 similarly sized *Stylophora pistillata* fragments (with a surface area of ~20 cm^2^) were collected from the Interuniversity Institute for Marine Sciences (IUI) coral nursery located in the Gulf of Aqaba (29°30′6.84“N, 34°55′4.09“E). Of the fragments, 30 were suspended from PVC stands using fine nylon threads and 10 were glued to plastic lids using underwater epoxy glue (Propoxy 20, Hercules Chemicals). All fragments underwent a six month acclimation period in shaded water tables with running seawater at ambient temperatures of 21–27 °C. In order to induce coral bleaching, 10 of the suspended fragments were transferred to a water table inside a darkened room supplied with similar running seawater. Within a month these fragments turned white and no algal chlorophyll signal was detected by PAM (Pulse Amplitude Modulation) fluorometry. Bleached corals were fed twice a week with one-day-old *Artemia salina* nauplii. Suspended fragments were used for batch experiments and upright fragments for the localized experiments.

### Experimental setup

Rapid H_2_O_2_ release from *S*. *pistillata* was studied in two setups: batch and localized experiments (Fig. S1). In the well mixed batch experiments we studied the accumulated response of the whole organism to non-localized stimuli. The stimuli tested with this simple setup were feeding and broadly administered physical and chemical stimuli (Supplementary Fig. S1a–c), in the presence and absence of calcium. In the non-mixed localized experiments we studied the coral’s response to local stimuli at high temporal resolution (Fig. S1d). To do this, we utilized narrow tubes to continuously collect water from the coral surface at the site of stimulation and at two control un-stimulated sites. In this finer setup, we tested the coral’s response to strong and gentle physical stimuli, increasing concentrations of chemical stimuli, and to coral mucus.

### Batch experiments

#### General design

Corals were suspended in 100 mL beakers and stirred gently (at 400 rpm; Fig. S1a). H_2_O_2_ concentrations were measured in 1 mL sub-samples retrieved from the beaker every 2–5 min, and transferred to cuvettes containing 20 μL of POHPPA reagent stock (see H_2_O_2_ measurements, below). Previous use of this experimental system had shown that stirring at 400 rpm with 1 cm long magnet, induces H_2_O_2_ release from *S. pistillata*^22^. Subsequently, H_2_O_2_ concentrations were monitored for 10 minutes prior to the administration of stimuli, and are referred as basal H_2_O_2_ release. In addition, control experiments were conducted with untreated corals (n = 6). All experiments were conducted under fluorescent laboratory lighting (~10 μmol quanta m^−2^ s^−1^, measured using Li-Cor LI-192 spherical quantum sensor ;YSI, Yellow Springs, USA) at a constant temperature (25 ± 1 °C). Coral fragments were usually used for experiments twice a week, and were never used more than once in the same day. Prior to the experiments, corals were allowed to recover from their handling for 30–60 min under running seawater. Experiments commenced once the coral polyps were extended.

#### Feeding experiments

One-day-old *Artemia salina* nauplii were gently added at a concentration of ~100 individuals per mL of seawater. Capture of prey was visually validated by the presence of paralyzed nauplii on the polyps (Fig. S1c). Experiments were performed on non-bleached (n = 10) and bleached corals (n = 9) in natural seawater and in artificial seawater with and without calcium. Probing for potential artefacts related to nauplii addition, we tested the accumulation of H_2_O_2_ due to intact or homogenized nauplii, and to paralyzed nauplii that were removed from the coral surface following capture but before predation (Supplemental S2).

#### Physical and Chemical stimuli and Cnidocyte counts

Fetal Bovine Serum (FBS; Biological Industries, Beit Haemek, Israel) was tested as a chemical stimulus for initiating rapid H_2_O_2_ release. FBS is a concentrated mixture of cellular compounds such as sugars, proteins and fatty acids that has previously been shown to induce cnidocyte discharge^40^. This experiment included three stages, each spanning ten minutes. First the basal release of H_2_O_2_ was measured, then a gentle physical stimulus was administered and H_2_O_2_ release was monitored, and finally a gentle physical stimulus and chemical stimulus was applied together followed by additional monitoring of H_2_O_2_ levels. The gentle physical stimulus was administered by gently brushing the smooth part of the Pasteur pipette against the coral’s polyps. The pressure applied was estimated as 9.2 ± 4.4 g/cm^2^ (by applying similar pressure on an analytical scale and accounting for the pipette’s surface area). The physical and chemical stimulus was applied similarly using a pipette coated in 50 μL of concentrated FBS. For the purpose of counting cnidocytes, an acid-cleaned human hair (6 cm long) was wrapped around the Pasteur pipettes and was removed for microscopic analysis after use. This method, similar to the one described by Watson *et al.*^41^ gives a crude estimate of the magnitude of cnidocyte discharge in response to the stimuli. The hair was placed on a microscopic slide and the number of discharged cnidocysts embedded in the hair was counted under a light microscope.

#### Physical stimulus

In nature, corals are exposed to multiple physical stressors, such as predation (e.g. by fish) or abrasion (e.g. by sand or silt during periods of intense wave or current action). We applied strong physical stimulus by sliding a glass Pasteur pipette along the coral fragment (Fig. S1b). We pressed and pulled the mouth of the pipette against the coral tissue five times in different areas of the coral. By pulling the pipette rather than pushing it, we avoided injury to the coral tissue by the pipette’s sharp edge (none such injuries were detected by the naked eye). We estimated the pressure applied by the pipette at a single point as 116 ± 28 g/cm^2^.

#### Calcium free conditions

To further link H_2_O_2_ release to prey capturing, we used Artificial Seawater (ASW) lacking calcium (Ca) as an alternative experimental medium. Ca free conditions are known to inhibit many metabolic pathways including cnidocyte discharge^42^. Artificial seawater with and without calcium was made according to Harrison *et al.*^43^. For the Ca-free ASW experiments, the coral fragments were pre-incubated for 15 min in Ca-free ASW to ensure complete removal of Ca leftovers.

### Localized experiments

Stony corals, such as *Stylophora pistillata*, are colonial organisms in which individual polyps are connected through the common coenosarc, potentially facilitating whole-colony behavior in response to localized stimulus. To test whether H_2_O_2_ is released only next to the site of stimulus or also further away in the colony, we utilized a container outfitted with narrow Teflon tubes (0.03 inch internal diameter) at three locations (Fig. 5; Fig. S1d). The tubes carried water by gravitation from the coral surface to cuvettes placed outside the container. The tube positions were adjustable and the opening to each tube was set at ~0.2 cm from the coral surface. One tube was placed next to the site of stimulus and the other two were placed ~2 cm away from it and served as controls (Fig. 5). Prior to the experiment, an upright-mounted coral fragment was placed on the container bottom, the tubes were adjusted and the coral was acclimatized in running seawater for ~1 hr. No stirring was applied during the experiment and care was taken to minimize turbulence while administering the localized stimulus. The tubes constantly transferred water at a rate of 2 mL min^−1^ from the coral surface to cuvettes that were replaced every 0.5–2 min. The experiments started with 8 minutes of background H_2_O_2_ measurements with water samples being collected every 2 min. After 8 minutes a stimulus was applied at one location over a small area of 10 mm^2^ adjacent to the opening of one of the tubes. Following the stimulus, H_2_O_2_ readings were taken every 30 seconds from all three sites. The stimuli applied using this setup were strong and gentle physical stimulus, two different doses of FBS, jets of water at different temperatures (100 μL of water at 10 °C, ambient temperature and 40 °C), and a biological stimulus (mucus from other *S. pistillata* fragments). The strong and gentle physical stimulus were applied over a small surface area as described above. Chemical stimulus was introduced by releasing 10 μl or 100 μl FBS on the coral tissue. To rule out the possibility that the H_2_O_2_ release was induced by the motion of fluid and not by the chemicals themselves, these experiments were repeated with similar volumes of filtered seawater. A biological stimulus was applied using 100 μl of coral mucus collected by air exposure of a separate *S. pistillata* colony.

### H_2_O_2_ measurements

H_2_O_2_ concentrations were measured using the POHPPA technique detailed in Shaked and Armoza-Zvuloni^21^. The POHPPA reagent stock, consisting of 0.25 mM POHPPA (4-hydroxyphenylacetic acid), 70 units mL^−1^ of horseradish peroxidase, and 0.25 M Tris at pH 8.8, was added to the samples at a 1:50 dilution. All stocks were kept on ice in the dark. The florescence of the dark-kept samples was read within 30 min of sampling using a Varian spectroflurometer (Cary Eclipse) set at an excitation of 315 ± 10 nm and an emission of 408 ± 10 nm. Calibration curves were run daily using filtered seawater spiked with freshly made H_2_O_2_ standards and catalase treated seawater as blanks. Calibrations were also conducted using artificial seawater (ASW), Calcium-free ASW or seawater with FBS when these mediums were used in the experiment. Reported concentrations were not corrected for reduction in water volume upon sampling, but nevertheless this has a negligible effect on the data presented (verified to be within 5% of variation) as well as on the statistical outcome.

### Statistical analyses

Statistical analyses were carried out using SPSS 20 statistical software. The data was tested for normality (Kolmogorov-Smirnov test) and homogeneity of variances (Levene’s test). When necessary, log or square root transformations were performed. In the batch experiments (Figs 1,2 and 4) we tested the change in H_2_O_2_ concentrations before and after applying the stimulus. For each section of the experiment, we calculated ΔH_2_O_2_ by subtracting the initial H_2_O_2_ measurement from the final H_2_O_2_ measurement and accounted for time differences between the two experimental sections. Differences in ΔH_2_O_2_ were tested using paired and independent samples t-tests. In bleached corals we tested the differences in H_2_O_2_ release before and during predation (Fig. 2b) using the nonparametric paired Wilcoxon’s test since the data did not match the homogeneity of variance criterion. To test the effect of gentle touch and chemical stimulus on H_2_O_2_ release (Fig. 3a,b) we used one way Repeated Measures ANOVA and Bonferroni’s test for post hoc comparison. In the localized experiments (Fig. 6), we tested the differences in H_2_O_2_ concentrations before applying the stimulus (at minute 8) and after (at minute 9.5 and 10) using one way Repeated Measures ANOVA and Bonferroni’s test for post hoc comparison.

## Additional Information

**How to cite this article**: Armoza-Zvuloni, R. *et al.* Rapid Hydrogen Peroxide release from the coral *Stylophora pistillata* during feeding and in response to chemical and physical stimuli. *Sci. Rep.* **6**, 21000; doi: 10.1038/srep21000 (2016).

## Supplementary Material

Supplementary Information

## References

[b1] Cartwright P. *et al.* Exceptionally preserved jellyfishes from the middle Cambrian. PLoS One 2(10) 1–7e1121 (2007). 10.1371/journal.pone.0001121 PMC2040521 17971881

[b2] Shinzato C. *et al.* Using the *Acropora digitifera* genome to understand coral responses to environmental change. Nature. 476, 320–323 (2011). 21785439 10.1038/nature10249

[b3] Frazão B., Vasconcelos V. & Antunes A. Sea anemone (Cnidaria, Anthozoa, Actiniaria) toxins: an overview. Mar. Drugs. 10, 1812–1851 (2012). 23015776 10.3390/md10081812 PMC3447340

[b4] Paul V. J. & Puglisi M. P. Chemical mediation of interactions among marine organisms. Nat. Prod. Rep. 21, 189–209 (2004). 15039843 10.1039/b302334f

[b5] Pawlik J. R. Marine invertebrate chemical defense. Chem. Rev. 93, 1911–1922 (1993).

[b6] Tardent P. The cnidarian cnidocyte, a high-tech cellular weaponry. Bioessay. 17, 351–362 (1995).

[b7] Nagai H. *et al.* Novel proteinaceous toxins from the Box Jellyfish (Sea Wasp). Carybdea rastoni. Biochem. Bioph. Res. Co. 275, 582–588 (2000). 10.1006/bbrc.2000.3353 10964707

[b8] Sher D., Fishman Y., Melamed-Book N., Zhang M. & Zlotkin E. Osmotically driven prey disintegration in the gastrovascular cavity of the green hydra by a pore-forming protein. FASEB J. 22, 207–221 (2008). 17679608 10.1096/fj.07-9133com

[b9] Moran Y. *et al.* Neurotoxin localization to ectodermal gland cells uncovers an alternative mechanism of venom delivery in sea anemones. Proc. Biol. Sci. 279, 1351–1358 (2012). 22048953 10.1098/rspb.2011.1731 PMC3282367

[b10] Gunthorpe L. & Cameron A. M. Widespread but variable toxicity in scleratinian corals. Toxicon 28, 1199–1219 (1990). 1979891 10.1016/0041-0101(90)90120-v

[b11] Apel K. & Hirt H. Reactive oxygen species: metabolism, oxidative stress, and signal transduction, Annu. Rev. Plant. Biol. 55, 373–399 (2004). 15377225 10.1146/annurev.arplant.55.031903.141701

[b12] Repetto M., Semprine J. & Boveris A. Lipid peroxidation: chemical mechanism, biological implications and analytical determinations. In Lipid Peroxidation, Catala A. (ed.), 1–28, Intech. (2012).

[b13] McDowell R. E., Amsler C. D., McClintock J. B. & Baker B. J. Reactive oxygen species as a marine grazing defense: H_2_O_2_ and wounded *Ascoseira mirabilis* both inhibit feeding by an amphipod grazer. J. Exp. Mar. Biol. Ecol. 458, 34–38 (2014).

[b14] Lamb C. & Dixon R. A. The oxidative burst in plant disease resistance. Annu. Rev. Plant. Phys. 48, 251–275 (1997). 10.1146/annurev.arplant.48.1.251. 15012264

[b15] Gustafsson M. S. M., Baird M. E. & Ralph P. J. Modeling photoinhibition-driven bleaching in Scleractinian coral as a function of light, temperature, and heterotrophy. Limnol. Oceanogr. 59, 603–622 (2014).

[b16] Rosenberg E., Koren O., Reshef L., Efrony R. & Rosenberg I. Z. The role of microorganisms in coral health, disease and evolution. Nat. Rev. Microbiol. 5, 355–362 (2007). 17384666 10.1038/nrmicro1635

[b17] Downs C. A. *et al.* Oxidative stress and seasonal coral bleaching. Free Radic. Biol. Med. 33, 533–543 (2002). 12160935 10.1016/s0891-5849(02)00907-3

[b18] Lesser M. P. Coral bleaching: causes and mechanisms. In: Coral Reefs: An Ecosystem In Transition, Dubinsky Z. & Stambler N. (eds.), 405–419, Springer, New York (2011).

[b19] Dunn S. R., Pernice M., Green K., Hoegh-Guldberg O. & Dove S. G. Thermal stress promotes host mitochondrial degradation in symbiotic cnidarians: are the batteries of the reef going to run out? PLoS ONE. 7, e39024, 10.1371/journal.pone.0039024 (2012). 22815696 PMC3398039

[b20] Saragosti E., Tchernov D., Katsir A. & Shaked Y. Extracellular production and degradation of superoxide in the coral *Stylophora pistillata* and cultured *Symbiodinium*. PLoS One. 5, e12508, 10.1371/journal.pone.0012508 (2010). 20856857 PMC2939047

[b21] Shaked Y. & Armoza-Zvuloni R. Dynamics of hydrogen peroxide in a coral reef: Sources and sinks. J. Geophys. Res.-Biogeo. 118, 1793–1801 (2013), 10.1002/2013JG002483.

[b22] Armoza-Zvuloni R. & Shaked Y. Release of hydrogen peroxide and antioxidants by the coral *Stylophora pistillata* to its external *milieu*. Biogeosciences. 11, 4587–4598 (2014). 10.5194/bg-11-4587-2014.

[b23] Shaked Y., Harris R. & Klein-Kedem N. Hydrogen peroxide photocycling in the Gulf of Aqaba, Red Sea. Environ. Sci. Technol. 44, 3238–3244 (2010). 20377174 10.1021/es902343y

[b24] Koehl M. A. R., Strother J. A., Reidenbach M. A., Koseff J. R. & Hadfield M. G. Individual-based model of larval transport to coral reefs in turbulent, wave-driven flow: behavioral responses to dissolved settlement inducer. Mar. Ecol. Prog. Ser. 335, 1–18 (2007).

[b25] Mass T., Genin A., Shavit U., Grinstein M. & Tchernov D. Flow enhances photosynthesis in marine benthic autotrophs by increasing the efflux of oxygen from the organism to the water. Proc. Natl. Acad. Sci. USA. 107, 2527–2531 (2010). 10.1073/pnas.0912348107. 20133799 PMC2823876

[b26] Shapiro O. *et al.* Vortical ciliary flows actively enhance mass transport in reef corals. Proc. Natl. Acad. Sci. USA. 111, 13391–13396 (2014). 10.1073/pnas.1323094111. 25192936 PMC4169935

[b27] Shashar N., Cohen Y. & Loya Y. Extreme diel fluctuations of oxygen in diffusive boundary layers surrounding stony corals. Biol. Bull. 185, 455–461 (1993). 10.2307/1542485 29300632

[b28] Mydlarz L. D. & Jacobs R. S. An inducible release of reactive oxygen radicals in four species of gorgonian corals. Mar. Freshw. Behav. Phy. 39, 143–152 (2006).

[b29] Santamaría A. *et al.* A venom extract from the sea anemone *Bartholomea annulata* produces haemolysis and lipid peroxidation in mouse erythrocytes. Toxicology 173, 221–228 (2002). 11960675 10.1016/s0300-483x(02)00035-5

[b30] Grzegorz B. *et al.* A pharmacological solution for a conspecific conflict: ROS-mediated territorial aggression in sea anemones. Toxicon. 51, 1038–1050 (2008). 18353415 10.1016/j.toxicon.2008.01.017

[b31] Ayed Y., Chayma B., Hayla A., Abid S. & Bacha H. Is cell death induced by nematocysts extract of medusa *Pelagia noctiluca* related to oxidative stress? Environ. Toxicol. 28, 498–506 (2011). 10.1002/tox.20740. 21809431

[b32] Wang T. *et al.* Lipid peroxidation is another potential mechanism besides pore-formation underlying hemolysis of tentacle extract from the jellyfish *Cyanea capillata*. Mar. Drugs. 11, 67–80 (2013). 10.3390/md11010067. 23303301 PMC3564158

[b33] Gough D. R. & Cotter T. G. Hydrogen peroxide: a Jekyll and Hyde signalling molecule. Cell. Death. Dis. 2, e213 (2011), 10.1038/cddis.2011.96. 21975295 PMC3219092

[b34] Munn C. B., Marchant H. K. & Moody A. J. Defences against oxidative stress in vibrios associated with corals. FEMS. Microbiol. Lett. 281, 58–63 (2008). 10.1111/j.1574-6968.2008.01073.x. 18279336

[b35] Benov L. & Fridovich I. Escherichia coli exhibits negative chemotaxis in gradients of hydrogen peroxide, hypochlorite, and N-chlorotaurine: Products of the respiratory burst of phagocytic cells. Proc. Natl. Acad. Sci. USA. 93, 4999–5002 (1996). 8643518 10.1073/pnas.93.10.4999 PMC39395

[b36] Voskuil M. I., Bartek I. L., Visconti K. & Schoolnik G. K. The response of Mycobacterium tuberculosis to reactive oxygen and nitrogen species. Front. Microbiol. 2, 105 (2011). 10.3389/fmicb.2011.00105. PMC3119406 21734908

[b37] Aggio J. F. & Derby C. D. Hydrogen peroxide and other components in the ink of sea hares are chemical defenses against predatory spiny lobsters acting through non-antennular chemoreceptors. J. Exp. Mar. Biol. Ecol. 363, 28–34 (2008).

[b38] Lesser M. P. Oxidative stress in marine environments: biochemistry and physiological ecology. Annu. Rev. Physiol. 68, 253–278 (2006). 10.1146/annurev.physiol.68.040104.110001. 16460273

[b39] Dixson D. L., Abrego D. & Hay M. E. Chemically mediated behavior of recruiting corals and fishes: A tipping point that may limit reef recovery. Science. 345, 892–897 (2014). 10.1126/science.1255057. 25146281 PMC4470392

[b40] Thorington G. U. & Hessinger D. A. Control of cnida discharge: I. evidence for two classes of chemoreceptor. Biol. Bull. 174, 163–171 (1988). 10.2307/1541539 29314979

[b41] Watson G. M., Mire P. & Kinler K. M. Mechanosensitivity in the model sea anemone *Nematostella vectensis*. Mar. Biol. 156, 2129–2137 (2009). 10.1007/s00227-009-1243-9.

[b42] Watson G. M. & Hessinger D. A. Evidence for calcium channels involved in regulating nematocyst discharge. Camp. Biochem. Physiol. 107, 473–481 (1994). 10.1016/0300-9629(94)90028-0 7909734

[b43] Harrison P. J., Waters R. E. & Taylor F. J. R. A broad spectrum artificial sea water medium for coastal and open ocean phytoplankton. J. Phycol. 16, 28–35 (1980).

