# Supplementary material for: Rapid Hydrogen Peroxide release from the coral Stylophora pistillata during feeding and in response to chemical and physical stimuli
Source: Sci Rep. 2016 Feb 15;6:21000. doi: 10.1038/srep21000 (PMC4753443; doi:10.1038/srep21000)
Supplement: Supplementary Information [file srep21000-s1.pdf]

Rapid Hydrogen Peroxide release from the coral *Stylophora pistillata* during feeding and in response to chemical and physical stimuli

Supplementary material

Rachel Armoza-Zvuloni, Avi Schneider, Daniel Sher and Yeala Shaked

List of contents:

- **Supplemental S1.** Setup for batch and localized experiments
- **Supplemental S2.** Controls for feeding experiments, validating that H_2O_2 release is from the coral and not the nauplii
- **Supplemental S3.** H_2O_2 release during feeding in bleached and non-bleached individual coral fragments
- **Supplemental S4.** Raw data of H_2O_2 release in response to chemical stimulus (FBS) in individual coral fragments
- **Supplemental S5.** H_2O_2 release in response to strong physical stimulus in individual coral fragments
- **Supplemental S6.** H_2O_2 release in response to strong physical stimulus in ASW and Ca free ASW in individual coral fragments
- **Supplemental S7.** H_2O_2 concentrations after coral removal, eliminating H_2O_2 production by enzymes released to the medium
- **Supplemental S8.** Controls for localized experiments, showing no response to hot and cold water
- **Supplemental S9.** Comparison of H_2O_2 release kinetics in the batch and localized experiments
- **Supplemental S10**. Calculating H_2O_2 concentration in the coral diffusive boundary layer

Supplemental S1. Setup for batch and localized experiments

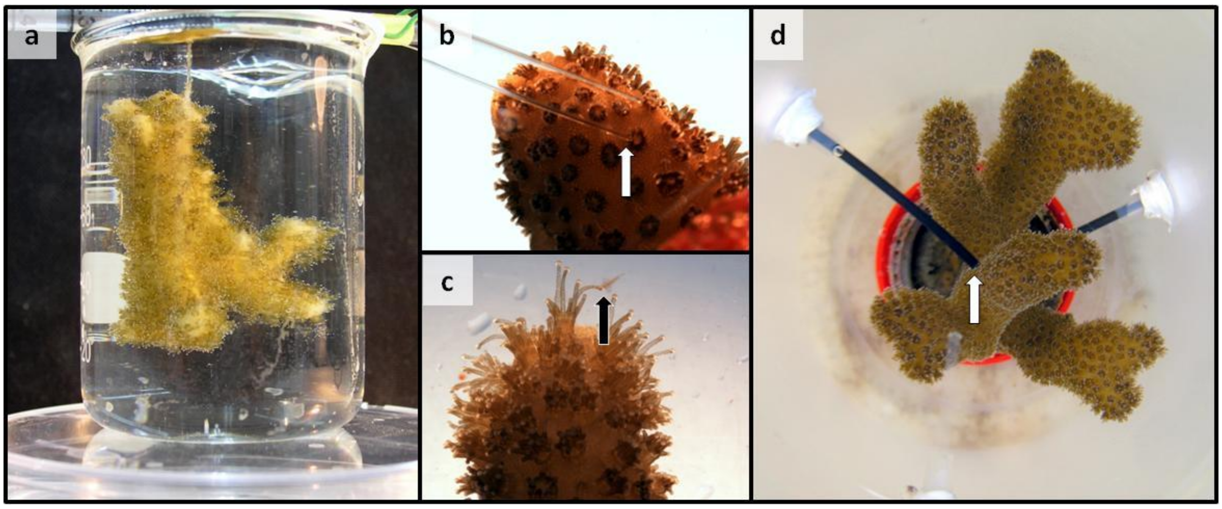

Fig. S1. In batch experiments (a) *Stylophora pistillata* coral fragments were suspended in 100 mL beakers and moderately stirred with magnetic bars. (b). Application of a physical stimulus with a Pasteur pipette caused immediate retraction of the coral polyps, but no visual damage was observed. (c). Feeding was tested with *Artemia salina* nauplii and prey capture was visually confirmed (arrow shows caught nauplius). (d). During localized experiments coral fragments (glued onto plastic lids) were placed upright and the openings of three sampling tubes were adjusted next to the coral surface. Water slowly dripping from the tubes was collected in cuvettes and measured for H_2O_2 every 0.5-2 min. The stimulus was carefully applied locally at one position only, trying to minimize stirring and turbulence.

Supplemental S2. Controls for feeding experiments, validating that H_2O_2 release is from the coral and not the *Artemia* nauplii

We made few control tests to verify that the H_2O_2 released in the feeding experiments originated from the coral and not from the *Artemia* nauplii. We were concerned with H_2O_2 release from live *Artemia* and/or H_2O_2 leakage from preyed-upon *Artemia*. We first added to seawater live *Artemia* at similar densities as in the experiments with the corals (100 individuals per mL seawater) and measured H_2O_2 concentrations over time. Upon artemia addition H_2O_2 slightly increased by ~20 nM, and then dropped back to the levels of *Artemia* -free seawater for the remaining 2-8 min (Fig. S2a). We then examined H_2O_2 release from *Artemia* crushed with a hand homogenizer (Fig. S2b). As before, we noted some increase (~20 nM) in H_2O_2 upon the addition of live *Artemia* nauplii (2nd & 3rd bars in Fig. S2b). The vigorous crushing of the *Artemia* resulted in release of additional 20 nM H_2O_2 initially, but this signal quickly declined (Fig. S2b times 1-3 min). Last we tested H_2O_2 release from *Artemia* that were captured by the coral, by gently removing them from the coral and re-suspending them in fresh seawater. We observed no H_2O_2 release from these *Artemia* in the 3 min we tested (Fig. S2c).

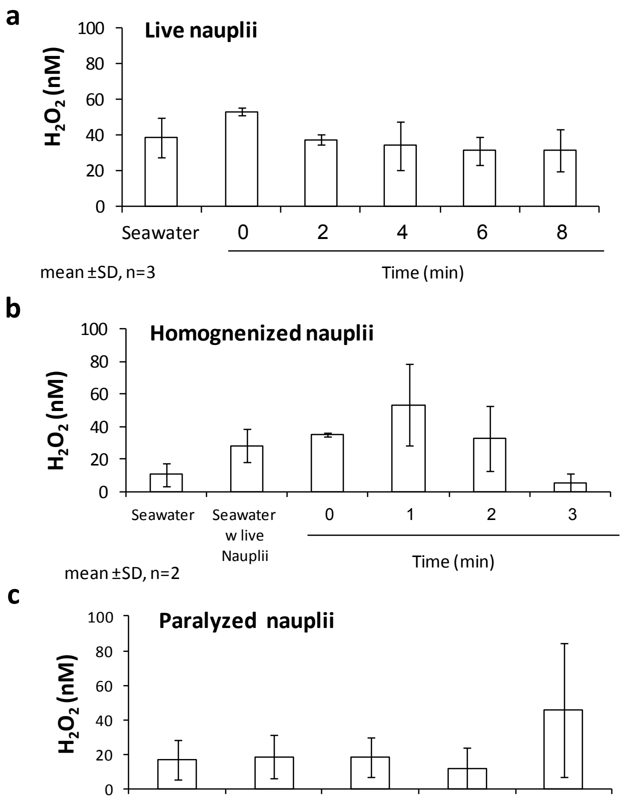

Fig. S2. H_2O_2 release from live, homogenized and paralyzed *Artemia*, as controls for H_2O_2 release in feeding experiments. (a) Addition of live *Artemia* to seawater. (b). Addition of homogenized *Artemia* to seawater. (c). Addition of *Artemia* previously paralyzed by the coral to new seawater. Note that the error bars on panel b represent ranges as n=2.

Supplemental S3. H_2O_2 release during feeding in bleached and non-bleached individual coral fragments

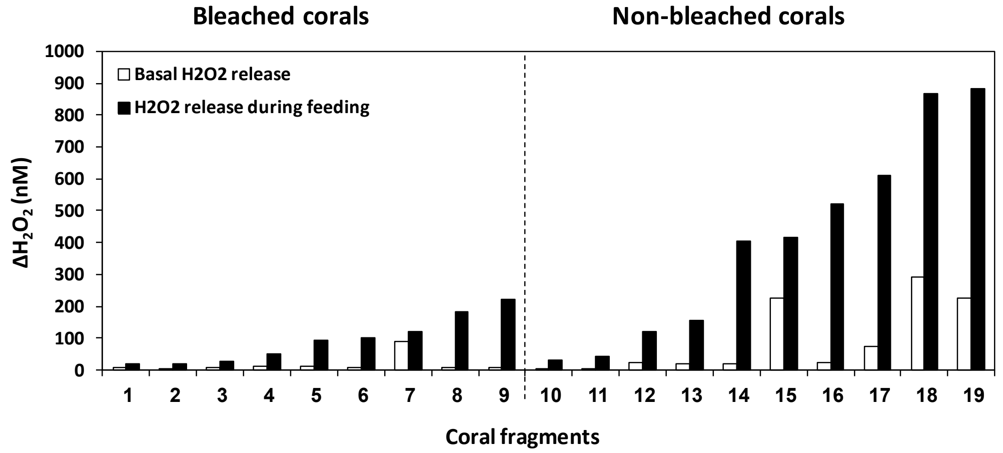

Fig. S3. Release of H_2O_2 during feeding by individual bleached (n=9) and non-bleached (n=10) fragments of *S. pistillata.* H_2O_2 concentrations were measured for ten minutes prior to food supply (white bars basal) and then for ten minutes during feeding (black bars). The bars represent the change in H_2O_2 concentration during these time intervals.

Supplemental S4. Raw data of H_2O_2 release in response to chemical stimulus (FBS)

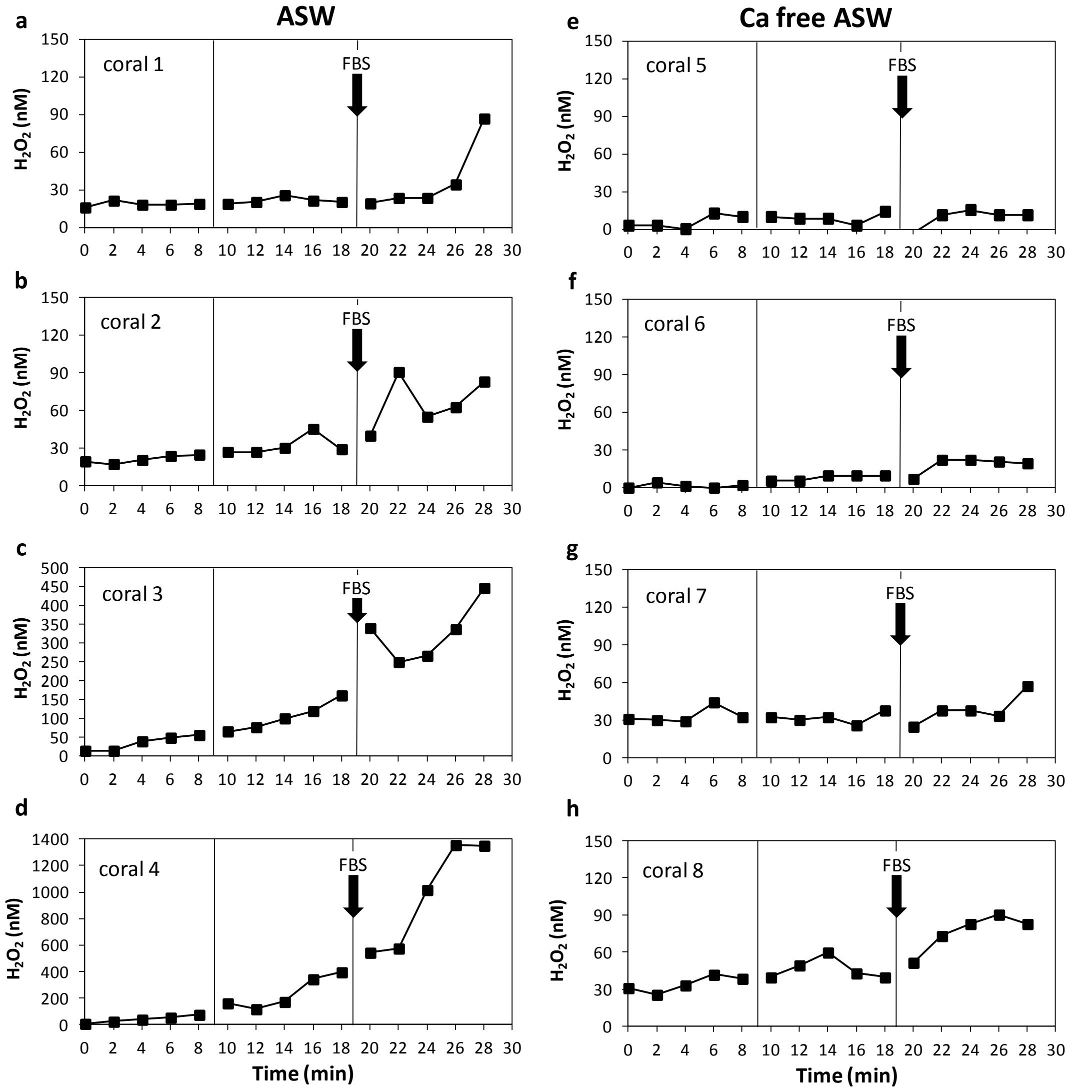

Fig. S4. H_2O_2 release from eight different *S. pistillata* corals triggered with Fetal Bovine Serum (FBS) in Artificial Seawater (ASW; a-d) and in Ca-free ASW (f-h). In the first 8 min of each experiment we recorded basal H_2O_2 release (due to stirring), then at 9 min gently touched the coral with Pastor pipette, and at 19 min repeated the gentle touch with FBS covered pipette.

Supplemental S5. H_2O_2 release in response to strong physical stimulus of individual coral fragments

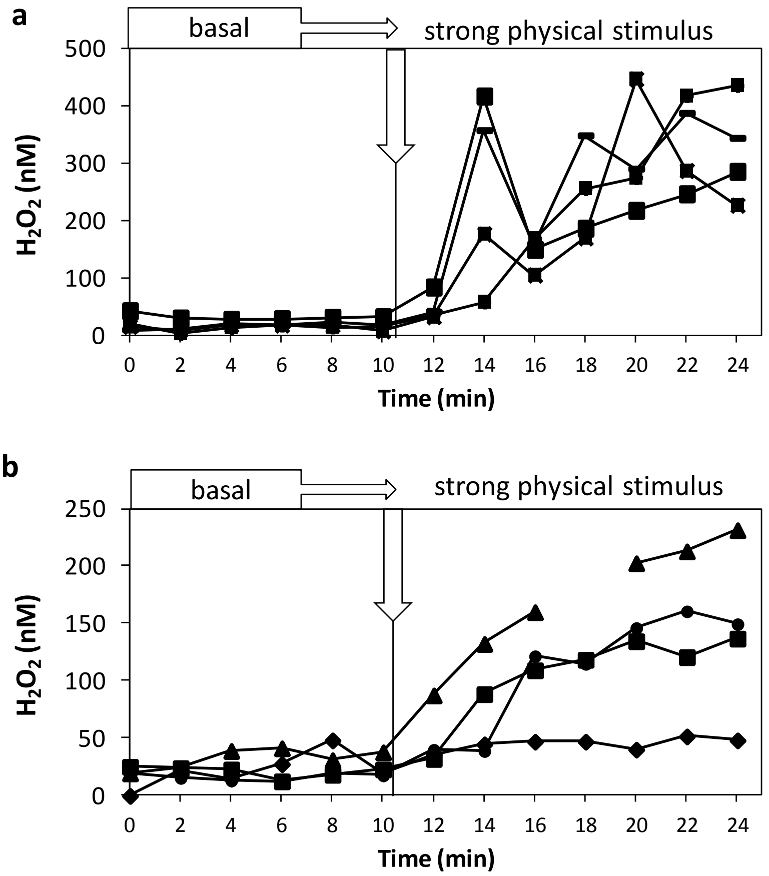

Fig. S5. Rapid H_2O_2 release from *S. pistillata* following strong physical stimulus. (a, b). Changes in H_2O_2 concentrations before applying the trigger (basal) and following physical stimulus in eight individual coral fragments. Note the differences in the Y-axes scale bar between panels a and b.

Supplemental S6. H_2O_2 release in response to strong physical stimulus in ASW and Ca free ASW in individual coral fragments

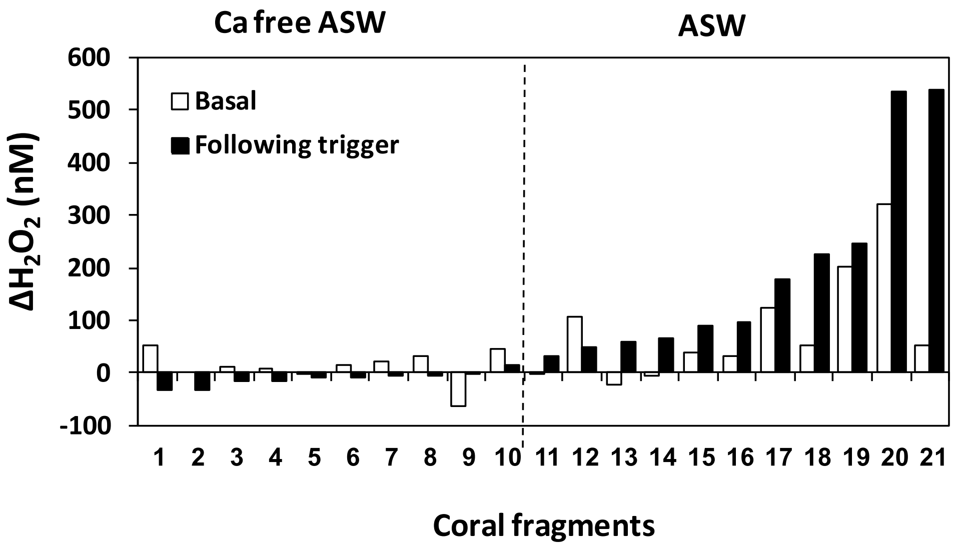

Fig. S6. Release of H_2O_2 by individual coral fragments following strong physical stimulus in Calcium free Artificial Seawater (Ca free ASW, n=10) and Artificial Seawater (ASW, n=11) as control. H_2O_2 concentrations were measured for ten minutes before triggering (white bars, basal) and for ten minutes following physical stimulus (black bars). The bars represent the change in H_2O_2 concentration during these time intervals.

Supplemental S7. H_2O_2 itself rather than a producing agent (e.g. compound or enzyme) is released from the coral to the medium

In search for the mechanisms of rapid H_2O_2 release we tested whether corals release enzymes to the medium that produce H_2O_2, by measuring H_2O_2 concentrations with time after coral removal. In the three tested corals, we first followed the basal H_2O_2 for 3 min. At 3 min we applied physical trigger and allowed 1.5 min for the presumed enzymes to be released to the medium. Then, at 4.5 min we removed 20 mL from each beaker and kept measuring H_2O_2 every 1.5 min for additional 10 min in the absence and presence of the coral. All corals responded to the physical trigger by releasing up to 1600 nM H_2O_2, but no H_2O_2 accumulated in the absence of the coral (Fig. S7).

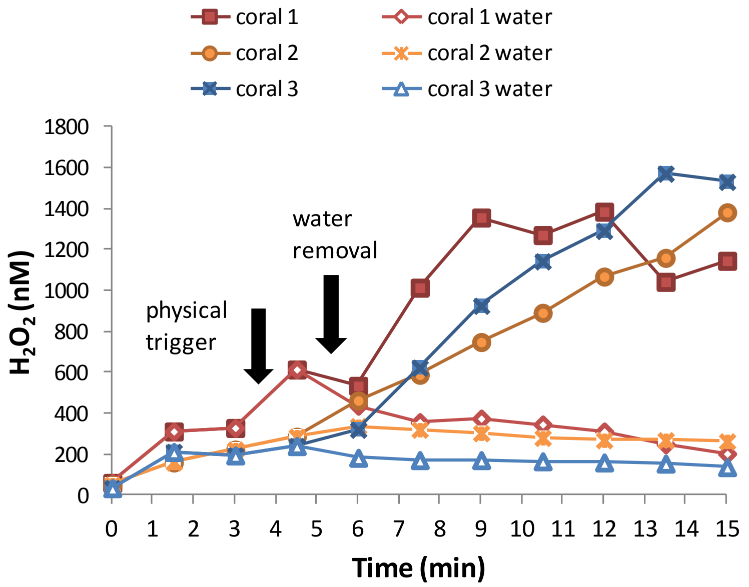

Fig. S7. Rapid H_2O_2 release from three *S. Pistillata* coral fragments triggered with physical stimulus and lack of H_2O_2 accumulation in water removed from these corals after the stimulus. The corals were triggered at 3 min and at 4.5 min aliquots were removed and followed with time for additional 10 min.

Supplemental S8. Controls for localized experiments, showing no response to hot and cold water

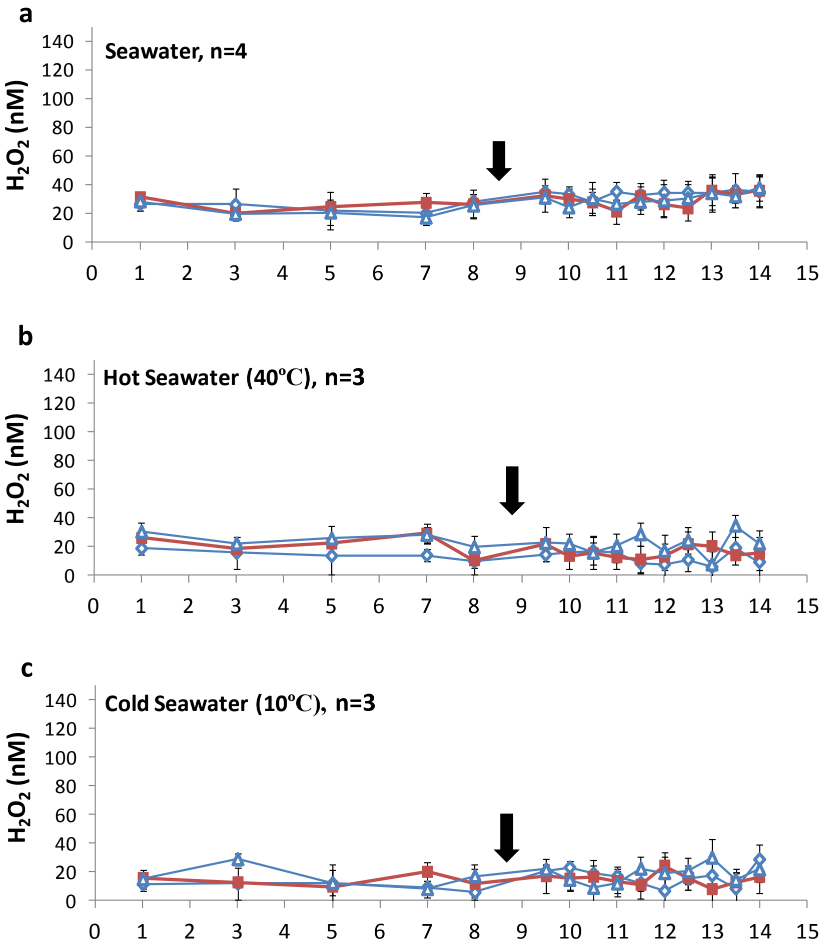

Fig. S8. Control treatments for the localized experiments, where 100 µl of seawater at different temperatures (a-25°C, b- 40°C, and c-10°C) were pipetted at the site of stimulus at 8.5 min. No response was measured from any of the treatments.

Supplemental S9. Comparison of H_2O_2 release kinetics in the batch and localized experiments.

The experimental systems we tested differ from one another in several aspects, such as water stirring (or lack of stirring), stimulus area (locally confined versus broad) and water removal or retention. The data obtained in each setup is typified by different H_2O_2 release kinetics: continues H_2O_2 accumulation in the batch versus abrupt rise in H_2O_2 followed by a gradual decline in the localized experiments. Below, we demonstrate by calculations that the two systems generate surprisingly similar H_2O_2 release kinetics, when accounting for the water removal (or lack of it).

In localized experiments, the H_2O_2 generated by the coral was efficiently removed by the narrow tube adjacent to the site of trigger, as no increase in H_2O_2 was seen in the other tubes (Fig. 5). Since all the removed water was collected in a series of cuvettes and H_2O_2 measured (Fig. S9a), we can calculate the moles of H_2O_2 released with time and sum them up. In Fig. S9 we present the accumulated H_2O_2 with time in units of nM, calculated for strong physical stimulus experiments in the localized setup (Fig. 6a). This exercise yielded a curve (Fig. S9b) that resembles that of batch experiments (e.g. Fig. 4)

In batch experiments, in turn, where H_2O_2 accumulated with time, and stirring contributed to low but steady H_2O_2 flux, H_2O_2 release kinetics is somewhat masked (Fig. S9c). Examining the change in H_2O_2 in each time interval, reveal a pattern that bare some resemblance to that of the localized experiments (Fig. S9d).

These calculations show that the response to the trigger is rapid and that it declines with time. We thus conclude that both experimental systems yield comparable H_2O_2 release kinetics.

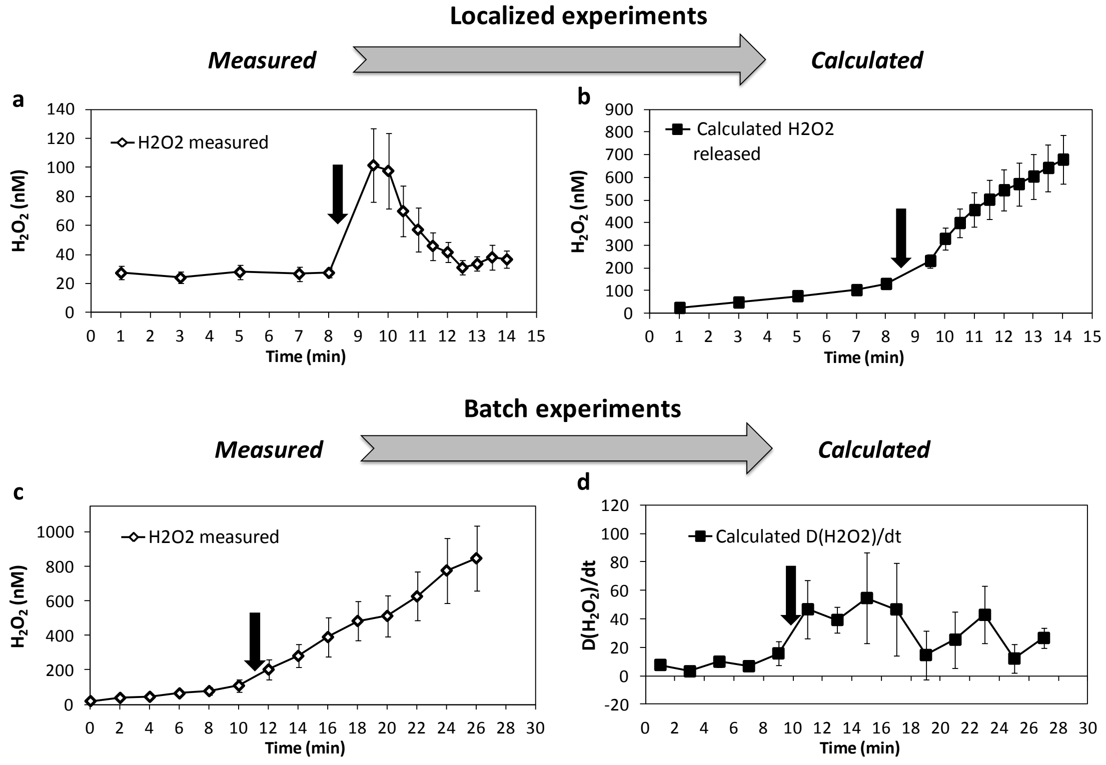

Fig. S9. Comparison of H_2O_2 release kinetics in the two setups. (a) Measured data from the localized experiments with strong physical stress (Fig. 6a). (b) Accumulated H_2O_2 release with time, calculated from the data in a. (c). Measured data from the predation batch experiments (Fig. 1c). (d). Calculated changes of H_2O_2 at small time intervals using the data in c.

Supplemental S10. Calculating H_2O_2 concentration in the coral diffusive boundary layer.

From the data obtained in the localized experiments we can attempt to estimate the concentration of H_2O_2 within the diffusive boundary layer (DBL). The rationale behind this calculation is that the coral-released signals we measured were probably diluted during collection in the tubes that cover a significantly larger volume than that of the DBL at the site of stimulus. For this calculation we sum up all the moles of H_2O_2 released and measured in the localized experiment and divide them by the small volume of DBL adjacent to the stimulus. The stimulus volume refers to the stimulus area times the diffusive boundary layer thickness, into which H_2O_2 was initially released. We assume for the sake of calculation that during the short period of active H_2O_2 release from the trigger site there are no (or low) diffusive losses from the boundary layer. We estimate the site of stimulus as 10 mm^2, the diffusive boundary layer as 2 mm and hence the overall volume into which H_2O_2 is released as 20 mm^3. We then convert the measured H_2O_2 concentrations in the discrete intervals following the stimulus into moles of H_2O_2, add them up, dividing this result by the volume of 20 mm^3. The concentration we obtain for the different experiments ranges between 1 and 44µM, with average ± SD values of 17±14.8 (n=9).
